# Penile Degloving Injury in an Adolescent with Congenital Hypothyroid

**DOI:** 10.1155/2012/464670

**Published:** 2012-03-25

**Authors:** Chauniqua Kiffin, Matthew Porcelli, Oksana Prychyna, Byron Pazmino, Daniel Pust, Joseph DeCostanza

**Affiliations:** Division of Trauma Services, Memorial Regional Hospital, 3501 Johnson Street, Hollywood, FL 33021, USA

## Abstract

This case follows a 17-year-old boy with congenital hypothyroidism who sustained penile skin avulsion secondary to a dog bite. Initially, an attempt was made to repair the wound using the avulsed skin flap itself as coverage. The repair was done immediately upon presentation to enhance the chances of adequate flap viability; however, the dorsal portion of the reconstruction necrosed within the following week. Ultimately a full thickness surgical graft (FTSG) repair was performed to ensure the most desirable outcome.

## 1. Introduction

Although industrial machinery, agricultural machine belts, and suction sexual stimulation devices are responsible for the majority of cases [1], animal and human bites do occur and carry additional risk factors. Specifically, an increased risk for developing infection and subsequent tissue necrosis exists, which worsens the operative outcome. Patients who experience genital injuries secondary to animal or human bites are often embarrassed and tend to present well after the initial injury. This delay in treatment lessens viability of tissue, reducing the potential for simple repair with the avulsed tissue flap [2].

## 2. Case Report

A 17-year-old boy of short stature was transferred to the trauma department after being attacked by a dog. He suffered extensive bites to the left lower extremity as well as the penis. The patient's heart rate fluctuated between 45 beats per minute (bpm) and 63 bpm; his blood pressure was 98 mmHg systolic and 67 mmhg diastolic. Upon admission, he was found to be profoundly hypothyroid with a thyroid-stimulating hormone of 483 *μ*IU/mL and a total thyroxin of 0.46 *μ*IU/mL. The patient's medical history was significant for congenital hypothyroidism, and he reported to have discontinued thyroid replacement therapy at the age of nine. An X-ray of the boy's hand and wrist revealed a bone age between 10 and 11 years. Throughout the patient's stay he demonstrated intermittent episodes of bradycardia (50 bpm) and hypotension (70 mmHg/40 mmHg). The patient was also discovered to have a mild to moderate pericardial effusion on transesophageal echocardiogram, presumably a sequela of the patient's untreated hypothyroidism.

**Figure 1:**
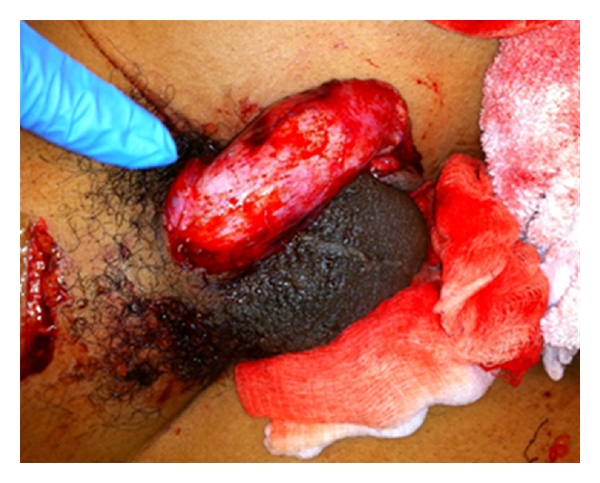


Upon examination, the patient was found to have an avulsion injury of the penis. Additional superficial lacerations were noted at the left thigh and over the symphysis pubis. The patient was taken to the operating room after the initial assessment. The penis was noted to be intact while the skin was entirely degloved. The skin covering the scrotum was intact (Figure 1). The presumably viable skin was rolled up to the head of the penis. Once unrolled, it was noted that the urethra was completely intact with no blood at the meatus. The skin was stretched the entirety of the penis to the base and reapproximated with interrupted sutures. Although there was no tension observed, the skin was reapproximated loosely to allow for some drainage due to the high risk of infection associated with dog bites. The superficial wounds to the thigh and pubis symphysis area had no penetration to the muscular fascia and were irrigated, debrided, and packed. Standard rabies postexposure prophylaxis was initiated at this time.

**Figure 2:**
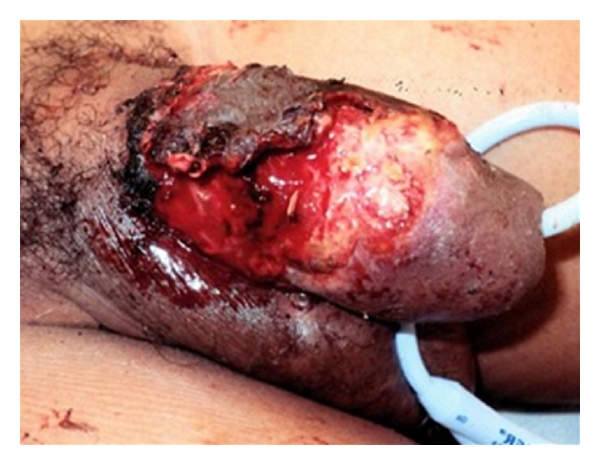


**Figure 3:**
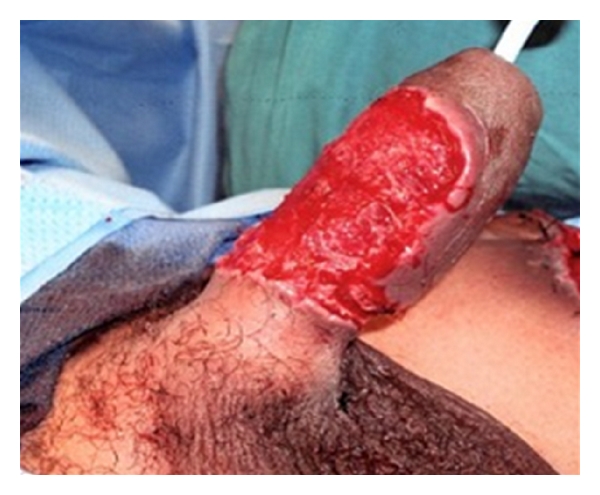


In the week following the repair, the penis remained edematous and a Foley catheter was left in place to prevent the urethra from closing shut. The dorsal aspect of penile skin blistered and necrosed; while the ventral aspect of the repair adhered well and appeared viable. The necrotic aspect of the penis was allowed to demarcate (Figure 2), which allowed for a specific debridement of the nonviable tissue (Figure 3).

**Figure 4:**
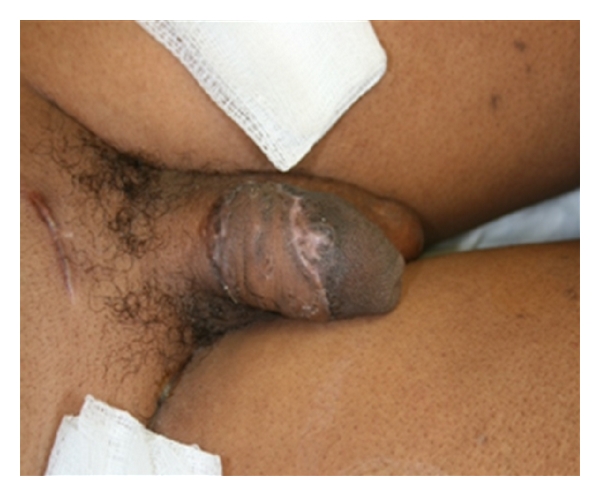
(FTSG 7 days post-op).

In the days following the dorsal debridement, the patient was taken to OR for graft repair with plastics. The penis remained swollen at the time of the operation, which was beneficial as it simulated an erection and allowed for a graft that would accommodate expansion. The wound was again irrigated and debrided, and a FTSG repair was performed (Figure 4). Right inguinal crease was used as the donor site, and the two edges were approximated leaving no area of exposure.

## 3. Discussion

The skin of the penis is considerably loose and elastic. This plasticity provides the penis with the ability to maintain both erect and flaccid states. Its unique composition leaves the penis susceptible to avulsion and degloving injuries [3]. The injury itself is not life threatening. The devastating effects stem from the sexually debilitating and psychologically damaging effects the injury manifests [4]. In reviewing the anatomy of the penis it is easy to recognize that degloving would be a common mechanism of injury to the penis. Starting distally, the glans penis remains in a fixed position, making it far less susceptible to a degloving injury. Loosened skin begins from the area immediately proximal to the coronal sulcus and extends until the base of the penis. The avulsed tissue consists of skin, superficial (dartos) fascia, and loose areolar tissue with its accompanying subcutaneous veins. It is this areolar tissue that forms a weak point between the two fascias creating a natural cleavage line. With shear force the loose skin is detached, revealing the intact bucks fascia. This signifies an unharmed corpora cavernosa, corpora spongiosum, and hence no damage to the urethra, dorsal artery, deep dorsal vein and nerve [5].

Since dartos fascia also covers the scrotum and is continuous with the colles fascia anteriorly, it is common to see avulsion of anterior portion of the scrotal skin together with penile skin. Testicular injury, however, is rare presumably owing to the cremasteric reflex saving the testes from involvement [6].

When dog bite is the mechanism of injury, the patient is at high risk for bacterial infection, with rates of 5–10%. Amoxicillin Clavulanate is the drug of choice for coverage of the most commonly involved organisms (*Staphylococcus, Streptococcus, Escherichia coli, and Pasteurella multocida*). Acceptable alternatives include oral Dicloxacillin, Cephalexin, Cefuroxime or Clindamycin, and Trimethoprim and Sulfamethoxazole. If the bite is from a human the wound is considered infected by definition and the wound should not be closed. An antibiotics regimen similar to dog bites can be used even though the bacterial organisms may differ [7].

An initial attempt should be made to close the wound with the patient's avulsed skin if at all possible. In our case this was attempted with relative success, salvaging a significant portion of the patient's avulsed skin. Since the patient was uncircumcised, there was even the hope that the foreskin could be utilized instead of a graft. With the lack of remaining viable tissue, this proved impractical and a graft was deemed necessary. Full thickness graft was chosen over split thickness graft (STSG) for a variety of potential advantages. These advantages are more elasticity, less anticipated contracture, and subsequent shortening or phimosis. FTSG is aesthetically desirable and can decrease the potential for painful erections. FTSG is more resistant to trauma, which is ideal considering the functionality of the penis. There was an added benefit to FTSG for this particular patient. The donor site needed additional wound care, which became a consideration due to the patient's special needs and anticipated noncompliance.

The only significant disadvantage to using a FTSG is a lower chance of graft survival compared to STSG but this drawback is balanced by the increased sensitivity to infection reported with STSG [8]. This is especially concerning in our patient where his congenital hypothyroidism could further delay wound healing and potentiate the risks of graft failure.

## 4. Conclusion

Penile skin avulsion is not uncommon. This traumatic injury is associated with an animal bite. Although this type of occurrence is not uncommon, animal bites do carry additional risk factors. The case illustrated a unique presentation of penile skin avulsion in a congenital hypothyroid male as a result of a dog bite. A FTSG graft was performed and yielded positive outcome.
